# Higher Coffee Consumption Is Associated With Reduced Cerebral Gray Matter Volume: A Mendelian Randomization Study

**DOI:** 10.3389/fnut.2022.850004

**Published:** 2022-03-17

**Authors:** Bing-Kun Zheng, Peng-Peng Niu

**Affiliations:** ^1^Neonatal Intensive Care Unit (NICU), The First Affiliated Hospital of Zhengzhou University, Zhengzhou, China; ^2^Department of Neurology, The First Affiliated Hospital of Zhengzhou University, Zhengzhou, China

**Keywords:** brain volume, magnetic resonance imaging, cerebral small vessel disease, Mendelian randomization (MR), coffee consumption

## Abstract

**Background:**

Recently published two-sample Mendelian randomization (MR) studies showed that genetically predicted coffee consumption may be associated with increased risk of Alzheimer’s disease and intracerebral hemorrhage but associated with a decreased risk of small vessel ischemic stroke. We aimed to investigate the effects of genetically predicted coffee consumption on magnetic resonance imaging (MRI) markers of cerebral small vessel disease and brain volume using the two-sample MR method.

**Methods:**

Twelve single nucleotide polymorphisms (SNPs) in up to 375,833 individuals were used as genetic instruments for cups consumed per day of coffee. Another four SNPs from an independent sample were used to perform the replication analysis. Three SNPs in up to 45,821 individuals were used as genetic instruments for high coffee consumption vs. low/no coffee consumption.

**Results:**

Mendelian randomization analysis showed that coffee consumption (cups/day) was inversely associated with gray matter volume (beta = −0.371, 95% CI = −0.596 to −0.147, *p* = 0.001). Replication analysis and multivariable analyses after adjusting for other risk factors confirmed the effect. High coffee consumption was also suggestively associated with decreased gray matter volume (beta = −0.061, 95% CI = −0.109 to −0.013, *p* = 0.013) compared with low/no coffee consumption. All analyses did not find an effect of coffee consumption on other outcomes including white matter hyperintensity volume, mean diffusivity, fractional anisotropy, brain microbleed, total brain volume, white matter volume, and hippocampus volume.

**Conclusion:**

This two-sample MR study showed that genetically predicted higher coffee consumption is causally associated with reduced gray matter volume of the brain.

## Introduction

Coffee is one of the leading consumed beverages worldwide. A study showed nearly half of the interviewed samples consumed at least one cup of coffee a day even during pregnancy (1). Therefore, the effects of coffee consumption on health outcomes are of interest and of importance.

A previous review of 201 meta-analyses of observational studies concluded that coffee consumption was more often associated with benefit than harm (2). Harmful associations were only found for the fracture in women and for the fetus (2). As for stroke, beneficial effects were also observed in observational studies (3, 4). A recent review found a significant inverse association between coffee consumption and risk of stroke (3). However, the effects of dementia were inconclusive (5, 6).

Since inferences from observational studies are often biased by confounding factors, a new method of two-sample Mendelian randomization (MR) has been widely used to assess the causality (7). Recently published studies using the two-sample MR method showed that genetically predicted coffee consumption may be associated with increased risk of Alzheimer’s disease and intracerebral hemorrhage but with a decreased risk of small vessel ischemic stroke (8, 9).

**FIGURE 1:**
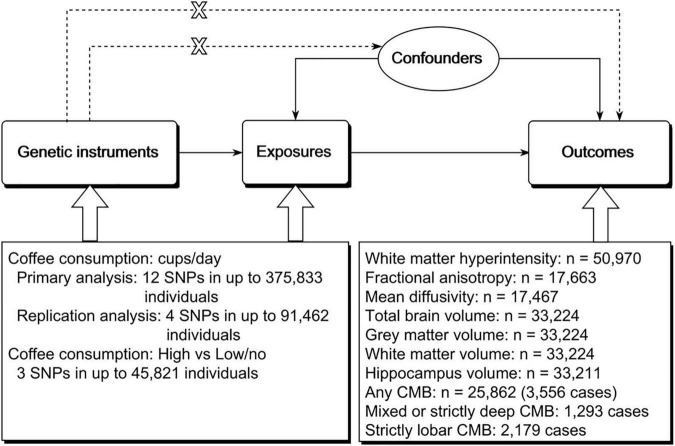
Schematic representation of the study design. The three assumptions of the two-sample MR study are as follows: genetic instrument is associated with exposure; genetic instrument is not associated with confounders; and genetic instrument does not affect outcome *via* pathways other than the exposure of interest. MR, Mendelian randomization; SNP, single nucleotide polymorphism; CMB, cerebral microbleed.

It has been well established that magnetic resonance imaging (MRI) markers of cerebral small vessel disease are associated with an increased risk of stroke, cognitive impairment, and dementia (10–14). In addition, brain volume is one of the surrogate measures of brain reserve, a concept that relates to cognitive function and Alzheimer’s disease (15). To provide more evidence regarding the effects of coffee consumption on cerebrovascular disease and cognitive function, we further investigated the effects of genetically predicted coffee consumption on MRI markers of cerebral small vessel disease and brain volume using the two-sample MR method (Figure 1).

## Materials and Methods

We followed the STROBE-MR statement to perform this study (16). Study-level summary data were used in this study. Ethical approval and informed consent were obtained in the original studies.

### Genetic Instruments

We used two phenotypes of coffee consumption. The first one is cups consumed per day. The other one is high coffee consumption vs. low/no coffee consumption. We selected independent single nucleotide polymorphisms (SNPs) (*r*^2^ < 0.01, ±500 kb) with genome-wide significance (*p* < 5 × 10^–8^) from previous studies (Supplementary Table 1).

Single nucleotide polymorphisms associated with cups consumed per day were obtained from a genome-wide association study (GWAS) of self-reported bitter and sweet beverage consumption among ∼370,000 participants of European ancestry (17). The discovery sample (*n* = 337,542) of the study was from the UK Biobank (18). The mean age of these individuals was 56.8 ± 8.0 years. Females accounted for 54.2% of these individuals. Coffee consumption in the UK Biobank was based on the question “How many cups of coffee do you drink each day (include decaffeinated coffee)?” Age, sex, body mass index, total energy, and top 20 principal components were adjusted. Independent SNPs with a *p*-value < 5 × 10^–6^ were followed-up in stage 2 for replication. Replication was carried out in three independent populations of European ancestry with a total of 39,984 individuals. The mean age of individuals in the replication sample was 54.2 years. There were 6,618 women (16.6%). Finally, a total of 15 SNPs associated with coffee consumption (*p* < 5 × 10^–8^) were reported by this study. The effects of these SNPs from the joint meta-analysis were similar to that of stage 1. In addition, all the *p*-values in stage 2 analysis were less than 0.01. We used effects from the joint analysis to perform the MR analysis. This could decrease the proportions of sample overlap between exposure and outcomes. We excluded three SNPs based on a linkage disequilibrium *r*^2^ of 0.01 and a window of ±500 kb. The total variance explained by the 12 SNPs was 0.005. The *F* statistics ranged from 30 to 713. The effects from this study were interpreted as a percentage change in consumption level per allele. We converted the effects to cups consumed per day.

Most of our outcome data were also from the UK Biobank. Sample overlap may bias the results of two-sample MR (19). However, a recent study showed that main two-sample MR methods can be safely used for one single large dataset than from large biobanks such as the UK Biobank (20). MR-Egger was not recommended due to the presence of bias. However, bias from MR-Egger was much reduced in the presence of very high variability in instrument strength across variants (*I*^2^_*GX*_ of 97%). The *I*^2^_*GX*_ of the 12 SNPs was 0.98. In addition, we calculated the bias using a web tool proposed by Burgess et al. (19).^1^ If the datasets of exposure and outcome are of different sizes, then the percentage over-lap was calculated based on the larger dataset (19). The calculated results showed that the biases were negligible (Supplementary Table 2).

We performed replication analysis using SNPs associated with cups consumed per day from another study. This study was a trans-ethnic meta-analysis of GWAS from 48 studies with up to 129,488 individuals (6.2% African Americans) (21). Analyses were adjusted for age and smoking status. Sex, case-control status, study-site, family structure, and/or study-specific principal components of population substructure were also adjusted (if possible). Replication was carried out for independent SNPs showing evidence for association (*p* < 1 × 10^–5^). Although the authors reported eight significant loci (10 SNPs), only four loci (6 SNPs) showed an inferred *p*-value smaller than 5 × 10^–8^. We used four independent SNPs (*r*^2^ < 0.01, ±500 kb) with effects from stage 1 (all European ancestry individuals) to perform the analysis. The *F* statistics ranged from 25 to 225.

Single nucleotide polymorphisms associated with high coffee consumption were also obtained from this study (21). High coffee consumption was defined as ≥4 cups per day in most original studies. Seventeen studies used a definition of ≥6 cups per day. Low/no coffee consumption was defined as <1 cup per day in most original studies. Fifteen studies used a definition of <2 cups per day. Four studies used a definition of never drinking. The authors reported five significant SNPs in up to 67,033 individuals. Effects from stage 1 were used to perform the MR analysis because the samples were all of the European ancestry individuals. We excluded two of them based on a linkage disequilibrium *r*^2^ of 0.01 and a window of ±500 kb. The *F* statistics of the three SNPs ranged from 30 to 132.

### Outcomes

#### White Matter Hyperintensity Volume

Summary data for white matter hyperintensity (WMH) was obtained from a large multi-ancestry meta-analysis of WMH-volume GWASs with 50,970 older individuals (95.1% European ancestry individuals) (22). The samples were from the Cohorts for Heart and Aging Research in Genomic Epidemiology (CHARGE) Consortium (*n* = 24,182) and the UK Biobank (*n* = 26,788) (18, 23). Individuals with a history of stroke (or MRI-defined brain infarcts involving the cortical gray matter) or other pathologies that may influence the measurement of WMH (e.g., brain tumor and head trauma) were excluded from analyses. Age, sex, principal components of population stratification, and intracranial volume were adjusted.

#### Mean Diffusivity and Fractional Anisotropy

Summary data for fractional anisotropy and mean diffusivity were obtained from a genetic study of MRI markers of small vessel disease (24). There were 17,663 individuals for fractional anisotropy and 17,467 individuals for mean diffusivity. Principal component analysis was performed on the fractional anisotropy and mean diffusivity of each of the 48 different brain tracts. The summary data were based on the first principal component. Individuals diagnosed with stroke or other diseases that could be associated with white matter damage were excluded from the analyses. Age, sex, genotyping array, the UK Biobank assessment center, the first 10 principal components, and MRI head motion indicators were adjusted. We did not use WMH data from this study because the full summary data for WMH were not provided.

#### Brain Microbleeds

Summary data for brain microbleeds (BMBs) were obtained from a meta-analysis of GWASs in 11 population-based cohort studies and three case-control or case-only stroke cohorts (25). BMBs were detected in 3,556 of the 25,862 participants (97.1% European ancestry individuals), of which 2,179 were strictly lobar and 1,293 were mixed. A total of 8,092 individuals were from the UK Biobank. Age and sex were adjusted in each included cohort.

#### Brain Volume

Summary data for brain volume were obtained from a GWAS of brain imaging-derived phenotypes in up to 33,224 individuals from the UK Biobank (26). The mean age of these individuals was 64.3 years. There were 17,411 women (52.4%). The data were quantile normalized, yielding a Gaussian distribution for each phenotype with mean = 0 and SD = 1. Confounders such as age, sex, head size, head motion, scanner table position, and imaging center were adjusted. Total brain volume (gray matter and white matter) (*n* = 33,224), gray matter volume (*n* = 33,224), white matter volume (*n* = 33,224), and hippocampus volume (left and right) (*n* = 33,211) were used to perform the MR analysis.

### Mendelian Randomization Analysis

We used the multiplicative random-effects inverse-variance weighted (IVW) method as the main method. It is the most efficient method when all genetic instruments are valid. It will return an unbiased estimate if the pleiotropy is null or balanced (27). The weighted median method and the weighted mode method were used for sensitivity analysis. These two methods will return unbiased estimates if some of the genetic instruments are invalid (27). Therefore, they are more robust to outliers and a small number of pleiotropic variants than the IVW method. Although substantial sample overlap exists between the exposure data and the outcome data, these three methods can be safely used on a single large dataset (20). The *I*^2^_*GX*_ from the 12 SNPs associated with cups consumed per day was 98%, which means that there was very high variability in instrument strength across variants. This suggests that the bias from the MR-Egger method due to sample overlap will be much reduced (20). Nevertheless, we did not use the MR-Egger method. We used the MR pleiotropy residual sum and outlier (MR-PRESSO) test to detect outliers (28). Since it is a variation based on the IVW method, it can be safely used.

We also performed the multivariable MR analysis to assess the direct effects of coffee consumption (29). We included the following common risk factors for cardiovascular disease: systolic blood pressure, type 2 diabetes, low-density lipoprotein cholesterol, body mass index, smoking, and alcohol drinking. Body mass index was not adjusted for primary data of cups per day because body mass index was already adjusted in the exposure summary data. Due to the same reason, we did not adjust smoking for high coffee consumption.

The MR analyses were performed using R (version 3.6.1) using packages of TwoSampleMR and MR-PRESSO (28, 30). A *p*-value < 0.002 [0.05/(2 × 11)] was considered statistically significant. A *p*-value < 0.05 was considered suggestively significant.

## Results

### Cups Consumed per Day

**FIGURE 2:**
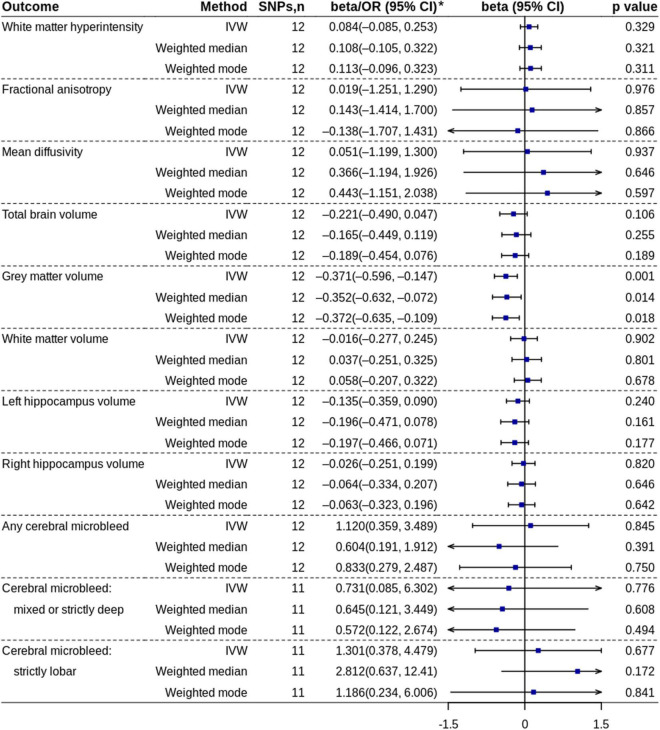
Mendelian randomization analyses of coffee consumption (cups/day) on each outcome. *ORs with 95% CIs are presented for cerebral microbleed outcomes. IVW, inverse variance-weighted; OR, odds ratio; SNP, single nucleotide polymorphism.

**FIGURE 3:**
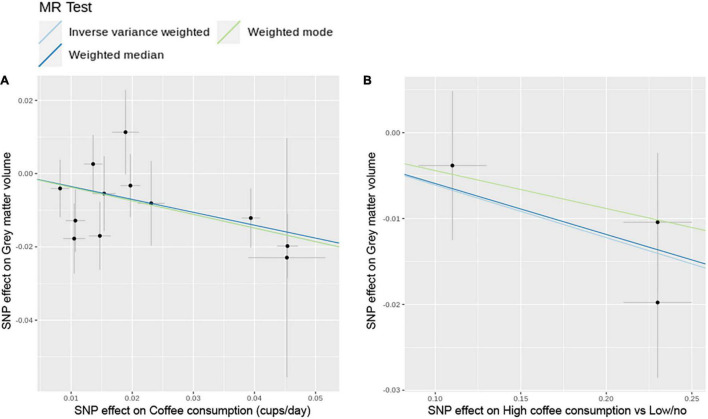
Scatter plots of SNP effect on coffee consumption and SNP effect on gray matter volume. **(A)** Cups per day of coffee consumption. **(B)** High coffee consumption vs. low/no coffee consumption. MR, Mendelian randomization; SNP, single nucleotide polymorphism.

**FIGURE 4:**
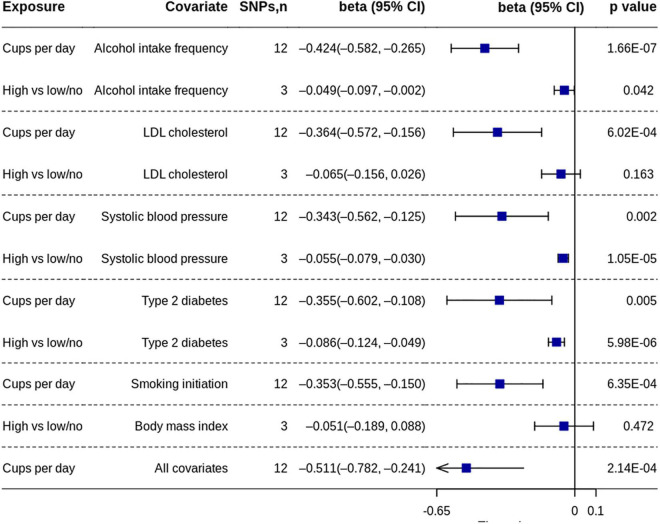
Multivariable MR analyses of coffee consumption on gray matter volume. MR, Mendelian randomization; SNP, single nucleotide polymorphism.

The MR analysis based on the IVW method showed that coffee consumption was inversely associated with gray matter volume (beta = −0.371, 95% CI = −0.596 to −0.147, *p* = 0.001) (Figures 2, 3). The MR analyses based on the weighted median method (beta = −0.352, 95% CI = −0.632 to −0.072, *p* = 0.014) and weighted mode method (beta = −0.372, 95% CI = −0.635 to −0.109, *p* = 0.018) showed similar results. MR-PRESSO test did not find outliers (global test *p*-value: 0.726). The leave-one-out plot showed that the effect was not driven by any single SNP (Supplementary Figure 1). The *p*-value of *Q*-statistic based on IVW was 0.652. Multivariable MR analysis adjusted for alcohol drinking, low-density lipoprotein cholesterol, systolic blood pressure, type 2 diabetes, and smoking one by one or altogether all showed similar results (Figure 4).

Replication analysis using the four SNPs from another independent study confirmed the effect of coffee consumption on gray matter volume (Supplementary Figure 2).

Both primary analyses and replication analyses did not find the effect of coffee consumption on other outcomes.

### High vs. Low/No Coffee Consumption

**FIGURE 5:**
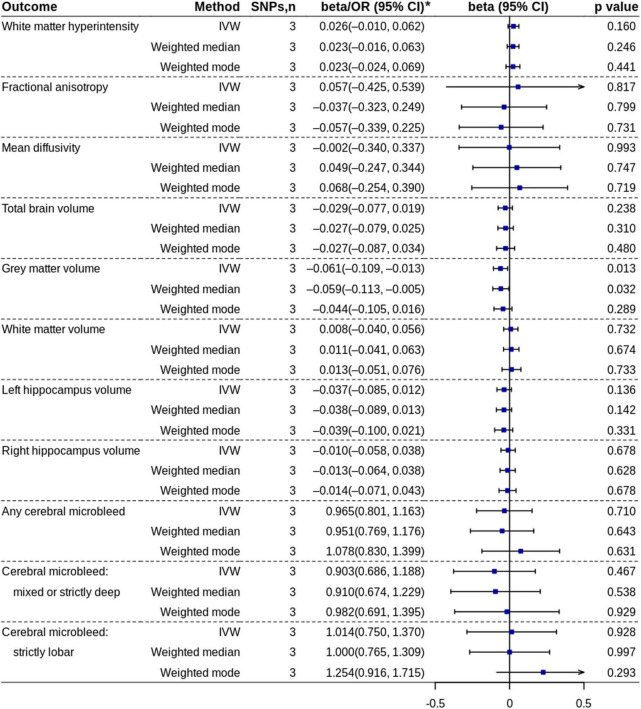
Mendelian randomization analyses of high coffee consumption vs. low/no coffee consumption. IVW, inverse variance-weighted; OR, odds ratio; SNP, single nucleotide polymorphism. * ORs with 95% CIs are presented for cerebral microbleed outcomes.

The MR analysis based on the IVW method showed that high coffee consumption was associated with decreased gray matter volume (beta = −0.061, 95% CI = −0.109 to −0.013, *p* = 0.013) (Figures 3, 5). The *p*-value of *Q*-statistic based on IVW was 0.691. MR analysis based on the weighted median method (beta = −0.059, 95% CI = −0.113 to −0.005, *p* = 0.032) showed a similar result. However, the effect based on the weighted mode method (beta = −0.044, 95% CI = −0.105 to 0.016, *p* = 0.289) attenuated to non-significant. The effects were significant when adjusting for alcohol drinking, systolic blood pressure, and type 2 diabetes, but they were not significant when adjusting for low-density lipoprotein cholesterol and body mass index (Figure 4). All other analyses did not find an effect of high coffee consumption on other outcomes.

## Discussion

This two-sample MR study showed that cups of coffee consumed per day were inversely associated with the gray matter volume of the brain. In addition, high coffee consumption was suggestively associated with decreased gray matter volume compared with low/no coffee consumption. We did not find evidence that genetically predicted coffee consumption was associated with WMH volume, mean diffusivity, fractional anisotropy, BMBs, total brain volume, white matter volume, or hippocampus volume.

A recently published observational study using data from the UK Biobank showed that coffee consumption was not associated with WMH volume, which was the same as the results of our study (31). However, the study showed that coffee consumption was inversely and significantly associated with total brain volume, gray matter volume, white matter volume, and hippocampal volume. Using genetic data from the UK Biobank with more MRI data samples, our MR study confirmed the causal effect of coffee consumption on gray matter volume. However, coffee consumption showed no causal effect on total brain volume, white matter volume, and hippocampal volume. Although our study showed coffee consumption trended toward a decrease in the total brain volume, this trend was mainly attributed to the effect of coffee consumption on the gray matter because the effect on the white matter was very close to null. Our finding was supported by a recently published small double-blind crossover randomized trial (32). This trial included 20 healthy male habitual caffeine consumers aged 18–35 years. This trial showed that 9-day administration of caffeine (3 doses of 150 mg/day) was associated with decreased gray matter volume compared with the placebo. For the association between coffee consumption and risk of cognitive disorders and Alzheimer’s disease, observational studies showed inconclusive results. An early dose-response meta-analysis showed a “J-shaped” association with the lowest risk of incident cognitive disorders at a daily consumption level of 1–2 cups of coffee (33). However, a subsequent dose-response meta-analysis did not support the non-linear relationship and did not find an association between coffee consumption and increased risk of overall dementia or Alzheimer’s disease (6). A meta-analysis of one-sample MR studies based on two genetic variants showed no effect of coffee intake on global cognition or memory (34). However, a recently published two-sample MR study showed that coffee consumption was associated with increased risk of Alzheimer’s disease (odds ratio = 1.31, 95% CI = 1.09–1.58, *p* = 0.005), but the effect attenuated to non-significant when adjusted for drinks per week (odds ratio = 1.20, 95% CI = 0.85–1.68, *p* = 0.305). Our study showed that coffee consumption can only affect gray matter volume and does not have an effect on hippocampal volume. In addition, we did not find evidence that coffee consumption was associated with MRI markers of cerebral small vessel disease. These may explain why the effects of coffee consumption on cognitive disorders and Alzheimer’s disease were not very robust in previous studies.

Although observational studies showed that coffee consumption was associated with a decreased risk of cardiovascular disease (including stroke) and cardiovascular mortality (2, 3), the effects were not fully supported by two-sample MR studies. Two two-sample MR studies showed that genetic predisposition to higher coffee consumption was not associated with any of the studied cardiovascular outcomes, except for a suggestive positive association for intracerebral hemorrhage after adjusting for body mass index and smoking (35, 36). Another two-sample MR study confirmed the harmful effects of coffee consumption on an intracerebral hemorrhage (8). However, this study and another two-sample MR study also showed weak suggestive evidence for a potential beneficial effect of coffee consumption on small vessel ischemic stroke (8, 9). Since small vessel ischemic stroke and intracerebral hemorrhage both belong to cerebral small vessel disease and shared similar risk factors (e.g., hypertension), it is interesting that coffee consumption showed totally opposite effects for them. Nevertheless, we did not find evidence that coffee consumption was associated with MRI markers of small vessel disease including WMH volume, mean diffusivity, fractional anisotropy, or BMBs. Therefore, taking into account both the weak suggestive evidence in previous studies and the findings of our study, the potential beneficial effect of coffee consumption on small vessel ischemic stroke found in previous studies should be interpreted with care, at least before being validated in an independent sample.

It has been suggested that the main components of coffee (i.e., caffeine and polyphenolic acids) may have antioxidant effects, anti-inflammatory effects, as well as neuroprotective effects through blocking the adenosine receptor (4, 37). In addition, *in vivo* studies showed that treatment with caffeine could prevent Aβ-induced cognitive impairment in mice (38, 39). The antioxidant and anti-inflammatory effects have been suggested to account for the potential protective effect of coffee consumption on the risk of small vessel ischemic stroke (9). However, intake of caffeine may temporarily increase the blood pressure in individuals with hypertension, which may lead to an increased risk of intracranial hemorrhage in hypertensive individuals (8, 40). A recently published study confirmed that coffee consumption is the main trigger factor for intracranial hemorrhage (41). Although caffeine could reinstate the adenosine-inhibited synaptic excitatory signaling in human neurons, it also could attenuate synaptic long-term potentials (42, 43). The impact of caffeine on long-term potentials might be partially responsible for the effects of coffee consumption on gray matter volume (31, 32).

This study has several limitations. First, there were substantial sample overlaps between primary data of cups per day and some outcomes. However, a recent study showed that main two-sample MR methods can be safely used for one single large dataset than from large biobanks such as the UK Biobank. In addition, we calculated the bias from sample overlap. The results showed that biases from sample overlap were negligible. Furthermore, replication analysis using an independent sample with no sample overlap supported the findings. Second, although primary analysis and replication analysis all showed coffee consumption (cups/day) was associated with decreased gray matter volume, the magnitude of the effects was quite different. This may be caused by the differences in included samples and analysis protocols. Third, although we included exposures of both cups per day and high coffee consumption vs. low/no coffee consumption, we could not analyze the potential non-linear relationship using summary data. Fourth, in the included studies, all types of coffee were treated the same to estimate total coffee consumption. Therefore, we could not perform the analysis stratified by types of coffee. It is uncertain whether different types of coffee have different influences for gray volume or not. Fifth, the duration of coffee consumption is certainly varied among individuals. For example, women are more likely to abstain from coffee during pregnancy and breastfeeding. However, the duration of coffee consumption was not adjusted in the GWASs of coffee consumption. Sixth, the power of the test was relatively low for some outcome measures. Finally, the samples included were mainly European ancestry individuals, which limits the generalizability of our findings for other populations.

## Conclusion

This two-sample MR study showed that genetically predicted coffee consumption was inversely associated with gray matter volume of the brain. We did not find evidence that genetically predicted coffee consumption was associated with WMH volume, mean diffusivity, fractional anisotropy, BMBs, total brain volume, white matter volume, or hippocampus volume.

## Data Availability Statement

The original contributions presented in the study are included in the article/Supplementary Material, further inquiries can be directed to the corresponding authors.

## Ethics Statement

Study-level summary data were used in the present study. Ethical approval and informed consent were obtained in the original studies. The ethics committee waived the requirement of written informed consent for participation.

## Author Contributions

B-KZ: data collection and roles, and writing—original draft. P-PN: conceptualization, data analysis, writing – review and editing, and supervision. Both authors contributed to the article and approved the submitted version.

## Conflict of Interest

The authors declare that the research was conducted in the absence of any commercial or financial relationships that could be construed as a potential conflict of interest.

## Publisher’s Note

All claims expressed in this article are solely those of the authors and do not necessarily represent those of their affiliated organizations, or those of the publisher, the editors and the reviewers. Any product that may be evaluated in this article, or claim that may be made by its manufacturer, is not guaranteed or endorsed by the publisher.

## Footnotes

1
https://sb452.shinyapps.io/overlap/
