# Supplementary material for: Higher Coffee Consumption Is Associated With Reduced Cerebral Gray Matter Volume: A Mendelian Randomization Study
Source: Front Nutr. 2022 Mar 17;9:850004. doi: 10.3389/fnut.2022.850004 (PMC8969755; doi:10.3389/fnut.2022.850004)
Supplement: Supplementary file 1 [file Data_Sheet_1.PDF]

eTable 1 Characteristics of genetic instruments

SNP	chr	pos	EA	OA	Exposure	Outcome	Exposure beta	se	eaf	p value	F statistic	Outcome beta	se
rs1057868	7	75615006	T	C	Exposure1	WMH	1.97	0.16	0.29	5.26E-33	152	0.0066	0.0063
rs10865548	2	631606	G	A	Exposure1	WMH	1.54	0.19	0.83	4.46E-15	66	0.0011	0.0078
rs1260326	2	27730940	C	T	Exposure1	WMH	1.36	0.15	0.61	2.62E-19	82	-0.0022	0.0064
rs1956218	14	33075243	G	A	Exposure1	WMH	0.82	0.15	0.56	3.62E-08	30	-0.0017	0.0058
rs2330783	22	24747031	G	T	Exposure1	WMH	4.53	0.63	0.99	1.57E-12	52	-0.0163	0.0241
rs2472297	15	75027880	T	C	Exposure1	WMH	4.54	0.17	0.27	5.19E-155	713	0.0039	0.0066
rs34060476	7	73037956	G	A	Exposure1	WMH	1.89	0.22	0.13	5.06E-18	74	0.0153	0.0088
rs4410790	7	17284577	C	T	Exposure1	WMH	3.94	0.15	0.63	5.59E-141	690	0.0059	0.0059
rs574367	1	177873210	T	G	Exposure1	WMH	1.05	0.18	0.21	8.06E-09	34	0.0026	0.0080
rs597045	11	56272114	A	T	Exposure1	WMH	1.07	0.16	0.69	6.62E-11	45	-0.0073	0.0070
rs66723169	18	57808978	A	C	Exposure1	WMH	1.47	0.18	0.23	9.88E-17	67	-0.0055	0.0068
rs73073176	7	17562952	C	T	Exposure1	WMH	2.31	0.22	0.87	5.56E-25	110	-0.0038	0.0087
rs1057868	7	75615006	T	C	Exposure1	FA	1.97	0.16	0.29	5.26E-33	152	0.0996	0.0464
rs10865548	2	631606	G	A	Exposure1	FA	1.54	0.19	0.83	4.46E-15	66	0.0262	0.0555
rs1260326	2	27730940	C	T	Exposure1	FA	1.36	0.15	0.61	2.62E-19	82	-0.0355	0.0431
rs1956218	14	33075243	G	A	Exposure1	FA	0.82	0.15	0.56	3.62E-08	30	-0.0016	0.0424
rs2330783	22	24747031	G	T	Exposure1	FA	4.53	0.63	0.99	1.57E-12	52	-0.2245	0.1787
rs2472297	15	75027880	T	C	Exposure1	FA	4.54	0.17	0.27	5.19E-155	713	0.0111	0.0479
rs34060476	7	73037956	G	A	Exposure1	FA	1.89	0.22	0.13	5.06E-18	74	0.0181	0.0628
rs4410790	7	17284577	C	T	Exposure1	FA	3.94	0.15	0.63	5.59E-141	690	-0.0385	0.0440
rs574367	1	177873210	T	G	Exposure1	FA	1.05	0.18	0.21	8.06E-09	34	-0.0757	0.0523
rs597045	11	56272114	A	T	Exposure1	FA	1.07	0.16	0.69	6.62E-11	45	0.0615	0.0463
rs66723169	18	57808978	A	C	Exposure1	FA	1.47	0.18	0.23	9.88E-17	67	0.0011	0.0505
rs73073176	7	17562952	C	T	Exposure1	FA	2.31	0.22	0.87	5.56E-25	110	0.0084	0.0624
rs1057868	7	75615006	T	C	Exposure1	MD	1.97	0.16	0.29	5.26E-33	152	-0.0607	0.0475
rs10865548	2	631606	G	A	Exposure1	MD	1.54	0.19	0.83	4.46E-15	66	-0.0902	0.0567
rs1260326	2	27730940	C	T	Exposure1	MD	1.36	0.15	0.61	2.62E-19	82	-0.0114	0.0440
rs1956218	14	33075243	G	A	Exposure1	MD	0.82	0.15	0.56	3.62E-08	30	0.0138	0.0434
rs2330783	22	24747031	G	T	Exposure1	MD	4.53	0.63	0.99	1.57E-12	52	0.1001	0.1826
rs2472297	15	75027880	T	C	Exposure1	MD	4.54	0.17	0.27	5.19E-155	713	0.0441	0.0489
rs34060476	7	73037956	G	A	Exposure1	MD	1.89	0.22	0.13	5.06E-18	74	0.0598	0.0642
rs4410790	7	17284577	C	T	Exposure1	MD	3.94	0.15	0.63	5.59E-141	690	-0.0002	0.0449
rs574367	1	177873210	T	G	Exposure1	MD	1.05	0.18	0.21	8.06E-09	34	-0.0092	0.0535
rs597045	11	56272114	A	T	Exposure1	MD	1.07	0.16	0.69	6.62E-11	45	-0.0323	0.0473
rs66723169	18	57808978	A	C	Exposure1	MD	1.47	0.18	0.23	9.88E-17	67	-0.0023	0.0516
rs73073176	7	17562952	C	T	Exposure1	MD	2.31	0.22	0.87	5.56E-25	110	0.0115	0.0638
rs1057868	7	75615006	T	C	Exposure1	TBV	1.97	0.16	0.29	5.26E-33	152	-0.0056	0.0086
rs10865548	2	631606	G	A	Exposure1	TBV	1.54	0.19	0.83	4.46E-15	66	-0.0100	0.0102
rs1260326	2	27730940	C	T	Exposure1	TBV	1.36	0.15	0.61	2.62E-19	82	0.0082	0.0079
rs1956218	14	33075243	G	A	Exposure1	TBV	0.82	0.15	0.56	3.62E-08	30	-0.0027	0.0078
rs2330783	22	24747031	G	T	Exposure1	TBV	4.53	0.63	0.99	1.57E-12	52	-0.0255	0.0327
rs2472297	15	75027880	T	C	Exposure1	TBV	4.54	0.17	0.27	5.19E-155	713	-0.0066	0.0088
rs34060476	7	73037956	G	A	Exposure1	TBV	1.89	0.22	0.13	5.06E-18	74	0.0209	0.0115
rs4410790	7	17284577	C	T	Exposure1	TBV	3.94	0.15	0.63	5.59E-141	690	-0.0068	0.0081
rs574367	1	177873210	T	G	Exposure1	TBV	1.05	0.18	0.21	8.06E-09	34	-0.0118	0.0096
rs597045	11	56272114	A	T	Exposure1	TBV	1.07	0.16	0.69	6.62E-11	45	-0.0219	0.0085
rs66723169	18	57808978	A	C	Exposure1	TBV	1.47	0.18	0.23	9.88E-17	67	-0.0140	0.0093
rs73073176	7	17562952	C	T	Exposure1	TBV	2.31	0.22	0.87	5.56E-25	110	-0.0130	0.0116
rs1057868	7	75615006	T	C	Exposure1	GMV	1.97	0.16	0.29	5.26E-33	152	-0.0033	0.0086
rs10865548	2	631606	G	A	Exposure1	GMV	1.54	0.19	0.83	4.46E-15	66	-0.0055	0.0102
rs1260326	2	27730940	C	T	Exposure1	GMV	1.36	0.15	0.61	2.62E-19	82	0.0026	0.0079
rs1956218	14	33075243	G	A	Exposure1	GMV	0.82	0.15	0.56	3.62E-08	30	-0.0040	0.0078
rs2330783	22	24747031	G	T	Exposure1	GMV	4.53	0.63	0.99	1.57E-12	52	-0.0229	0.0327
rs2472297	15	75027880	T	C	Exposure1	GMV	4.54	0.17	0.27	5.19E-155	713	-0.0198	0.0088
rs34060476	7	73037956	G	A	Exposure1	GMV	1.89	0.22	0.13	5.06E-18	74	0.0113	0.0115
rs4410790	7	17284577	C	T	Exposure1	GMV	3.94	0.15	0.63	5.59E-141	690	-0.0121	0.0081
rs574367	1	177873210	T	G	Exposure1	GMV	1.05	0.18	0.21	8.06E-09	34	-0.0177	0.0096
rs597045	11	56272114	A	T	Exposure1	GMV	1.07	0.16	0.69	6.62E-11	45	-0.0128	0.0085
rs66723169	18	57808978	A	C	Exposure1	GMV	1.47	0.18	0.23	9.88E-17	67	-0.0170	0.0093
rs73073176	7	17562952	C	T	Exposure1	GMV	2.31	0.22	0.87	5.56E-25	110	-0.0081	0.0116
rs1057868	7	75615006	T	C	Exposure1	WMV	1.97	0.16	0.29	5.26E-33	152	-0.0061	0.0086
rs10865548	2	631606	G	A	Exposure1	WMV	1.54	0.19	0.83	4.46E-15	66	-0.0098	0.0102
rs1260326	2	27730940	C	T	Exposure1	WMV	1.36	0.15	0.61	2.62E-19	82	0.0096	0.0079
rs1956218	14	33075243	G	A	Exposure1	WMV	0.82	0.15	0.56	3.62E-08	30	-0.0003	0.0078
rs2330783	22	24747031	G	T	Exposure1	WMV	4.53	0.63	0.99	1.57E-12	52	-0.0197	0.0327
rs2472297	15	75027880	T	C	Exposure1	WMV	4.54	0.17	0.27	5.19E-155	713	0.0073	0.0088
rs34060476	7	73037956	G	A	Exposure1	WMV	1.89	0.22	0.13	5.06E-18	74	0.0209	0.0115
rs4410790	7	17284577	C	T	Exposure1	WMV	3.94	0.15	0.63	5.59E-141	690	-0.0002	0.0081
rs574367	1	177873210	T	G	Exposure1	WMV	1.05	0.18	0.21	8.06E-09	34	-0.0030	0.0096

rs597045	11	56272114	A	T	Exposure1	WMV	1.07	0.16	0.69	6.62E-11	45	-0.0207	0.0085
rs66723169	18	57808978	A	C	Exposure1	WMV	1.47	0.18	0.23	9.88E-17	67	-0.0050	0.0093
rs73073176	7	17562952	C	T	Exposure1	WMV	2.31	0.22	0.87	5.56E-25	110	-0.0134	0.0116
rs1057868	7	75615006	T	C	Exposure1	left HV	1.97	0.16	0.29	5.26E-33	152	-0.0007	0.0086
rs10865548	2	631606	G	A	Exposure1	left HV	1.54	0.19	0.83	4.46E-15	66	-0.0103	0.0102
rs1260326	2	27730940	C	T	Exposure1	left HV	1.36	0.15	0.61	2.62E-19	82	-0.0017	0.0079
rs1956218	14	33075243	G	A	Exposure1	left HV	0.82	0.15	0.56	3.62E-08	30	0.0107	0.0078
rs2330783	22	24747031	G	T	Exposure1	left HV	4.53	0.63	0.99	1.57E-12	52	-0.0126	0.0327
rs2472297	15	75027880	T	C	Exposure1	left HV	4.54	0.17	0.27	5.19E-155	713	-0.0104	0.0088
rs34060476	7	73037956	G	A	Exposure1	left HV	1.89	0.22	0.13	5.06E-18	74	0.0152	0.0115
rs4410790	7	17284577	C	T	Exposure1	left HV	3.94	0.15	0.63	5.59E-141	690	-0.0069	0.0081
rs574367	1	177873210	T	G	Exposure1	left HV	1.05	0.18	0.21	8.06E-09	34	-0.0111	0.0096
rs597045	11	56272114	A	T	Exposure1	left HV	1.07	0.16	0.69	6.62E-11	45	-0.0012	0.0085
rs66723169	18	57808978	A	C	Exposure1	left HV	1.47	0.18	0.23	9.88E-17	67	0.0153	0.0093
rs73073176	7	17562952	C	T	Exposure1	left HV	2.31	0.22	0.87	5.56E-25	110	-0.0122	0.0116
rs1057868	7	75615006	T	C	Exposure1	right HV	1.97	0.16	0.29	5.26E-33	152	0.0044	0.0086
rs10865548	2	631606	G	A	Exposure1	right HV	1.54	0.19	0.83	4.46E-15	66	-0.0021	0.0102
rs1260326	2	27730940	C	T	Exposure1	right HV	1.36	0.15	0.61	2.62E-19	82	-0.0055	0.0079
rs1956218	14	33075243	G	A	Exposure1	right HV	0.82	0.15	0.56	3.62E-08	30	0.0043	0.0078
rs2330783	22	24747031	G	T	Exposure1	right HV	4.53	0.63	0.99	1.57E-12	52	-0.0240	0.0327
rs2472297	15	75027880	T	C	Exposure1	right HV	4.54	0.17	0.27	5.19E-155	713	-0.0018	0.0088
rs34060476	7	73037956	G	A	Exposure1	right HV	1.89	0.22	0.13	5.06E-18	74	0.0045	0.0115
rs4410790	7	17284577	C	T	Exposure1	right HV	3.94	0.15	0.63	5.59E-141	690	-0.0039	0.0081
rs574367	1	177873210	T	G	Exposure1	right HV	1.05	0.18	0.21	8.06E-09	34	0.0107	0.0096
rs597045	11	56272114	A	T	Exposure1	right HV	1.07	0.16	0.69	6.62E-11	45	0.0080	0.0085
rs66723169	18	57808978	A	C	Exposure1	right HV	1.47	0.18	0.23	9.88E-17	67	-0.0032	0.0093
rs73073176	7	17562952	C	T	Exposure1	right HV	2.31	0.22	0.87	5.56E-25	110	-0.0016	0.0116
rs1057868	7	75615006	T	C	Exposure1	Any BMB	1.97	0.16	0.29	5.26E-33	152	-0.0148	0.0313
rs10865548	2	631606	G	A	Exposure1	Any BMB	1.54	0.19	0.83	4.46E-15	66	0.0158	0.0376
rs1260326	2	27730940	C	T	Exposure1	Any BMB	1.36	0.15	0.61	2.62E-19	82	0.0485	0.0284
rs1956218	14	33075243	G	A	Exposure1	Any BMB	0.82	0.15	0.56	3.62E-08	30	-0.0203	0.0277
rs2330783	22	24747031	G	T	Exposure1	Any BMB	4.53	0.63	0.99	1.57E-12	52	0.0699	0.1369
rs2472297	15	75027880	T	C	Exposure1	Any BMB	4.54	0.17	0.27	5.19E-155	713	-0.0441	0.0342
rs34060476	7	73037956	G	A	Exposure1	Any BMB	1.89	0.22	0.13	5.06E-18	74	0.1312	0.0442
rs4410790	7	17284577	C	T	Exposure1	Any BMB	3.94	0.15	0.63	5.59E-141	690	0.0273	0.0290
rs574367	1	177873210	T	G	Exposure1	Any BMB	1.05	0.18	0.21	8.06E-09	34	-0.0461	0.0347
rs597045	11	56272114	A	T	Exposure1	Any BMB	1.07	0.16	0.69	6.62E-11	45	-0.0344	0.0314
rs66723169	18	57808978	A	C	Exposure1	Any BMB	1.47	0.18	0.23	9.88E-17	67	0.0311	0.0331
rs73073176	7	17562952	C	T	Exposure1	Any BMB	2.31	0.22	0.87	5.56E-25	110	-0.0427	0.0442
rs1057868	7	75615006	T	C	Exposure1	BMB lobar	1.97	0.16	0.29	5.26E-33	152	-0.0287	0.0391
rs10865548	2	631606	G	A	Exposure1	BMB lobar	1.54	0.19	0.83	4.46E-15	66	0.0185	0.0483
rs1260326	2	27730940	C	T	Exposure1	BMB lobar	1.36	0.15	0.61	2.62E-19	82	0.0307	0.0357
rs1956218	14	33075243	G	A	Exposure1	BMB lobar	0.82	0.15	0.56	3.62E-08	30	-0.0149	0.0345
rs2472297	15	75027880	T	C	Exposure1	BMB lobar	4.54	0.17	0.27	5.19E-155	713	-0.0419	0.0426
rs34060476	7	73037956	G	A	Exposure1	BMB lobar	1.89	0.22	0.13	5.06E-18	74	0.1358	0.0558
rs4410790	7	17284577	C	T	Exposure1	BMB lobar	3.94	0.15	0.63	5.59E-141	690	0.0537	0.0365
rs574367	1	177873210	T	G	Exposure1	BMB lobar	1.05	0.18	0.21	8.06E-09	34	-0.0627	0.0444
rs597045	11	56272114	A	T	Exposure1	BMB lobar	1.07	0.16	0.69	6.62E-11	45	0.0140	0.0400
rs66723169	18	57808978	A	C	Exposure1	BMB lobar	1.47	0.18	0.23	9.88E-17	67	0.0170	0.0415
rs73073176	7	17562952	C	T	Exposure1	BMB lobar	2.31	0.22	0.87	5.56E-25	110	-0.0501	0.0571
rs1057868	7	75615006	T	C	Exposure1	BMB deep	1.97	0.16	0.29	5.26E-33	152	0.0067	0.0474
rs10865548	2	631606	G	A	Exposure1	BMB deep	1.54	0.19	0.83	4.46E-15	66	-0.0305	0.0578
rs1260326	2	27730940	C	T	Exposure1	BMB deep	1.36	0.15	0.61	2.62E-19	82	0.1244	0.0444
rs1956218	14	33075243	G	A	Exposure1	BMB deep	0.82	0.15	0.56	3.62E-08	30	-0.0673	0.0428
rs2472297	15	75027880	T	C	Exposure1	BMB deep	4.54	0.17	0.27	5.19E-155	713	-0.0583	0.0517
rs34060476	7	73037956	G	A	Exposure1	BMB deep	1.89	0.22	0.13	5.06E-18	74	0.1459	0.0711
rs4410790	7	17284577	C	T	Exposure1	BMB deep	3.94	0.15	0.63	5.59E-141	690	0.0076	0.0448
rs574367	1	177873210	T	G	Exposure1	BMB_deep	1.05	0.18	0.21	8.06E-09	34	-0.0196	0.0537
rs597045	11	56272114	A	T	Exposure1	BMB_deep	1.07	0.16	0.69	6.62E-11	45	-0.1257	0.0476
rs66723169	18	57808978	A	C	Exposure1	BMB_deep	1.47	0.18	0.23	9.88E-17	67	0.0624	0.0510
rs73073176	7	17562952	C	T	Exposure1	BMB_deep	2.31	0.22	0.87	5.56E-25	110	-0.1181	0.0686
rs17685	7	75454041	A	G	Exposure1_2	WMH	0.07	0.01	0.29	9.06E-14	49	0.0074	0.0072
rs2472297	15	72814933	T	C	Exposure1_2	WMH	0.15	0.01	0.24	6.45E-47	225	0.0039	0.0066
rs4410790	7	17251102	T	C	Exposure1_2	WMH	-0.14	0.01	0.37	1.48E-57	196	-0.0059	0.0059
rs7800944	7	72673793	T	C	Exposure1_2	WMH	-0.05	0.01	0.72	7.82E-09	25	-0.0146	0.0064
rs17685	7	75454041	A	G	Exposure1_2	FA	0.07	0.01	0.29	9.06E-14	49	0.1177	0.0467
rs2472297	15	72814933	T	C	Exposure1_2	FA	0.15	0.01	0.24	6.45E-47	225	0.0111	0.0479
rs4410790	7	17251102	T	C	Exposure1 2	FA	-0.14	0.01	0.37	1.48E-57	196	0.0385	0.0440
rs7800944	7	72673793	T	C	Exposure1 2	FA	-0.05	0.01	0.72	7.82E-09	25	-0.0366	0.0468
rs17685	7	75454041	A	G	Exposure1 2	MD	0.07	0.01	0.29	9.06E-14	49	-0.0734	0.0478
rs2472297	15	72814933	T	C	Exposure1 2	MD	0.15	0.01	0.24	6.45E-47	225	0.0441	0.0489
rs4410790	7	17251102	T	C	Exposure1 2	MD	-0.14	0.01	0.37	1.48E-57	196	0.0002	0.0449

rs7800944	7	72673793	T	C	Exposure1_2	MD	-0.05	0.01	0.72	7.82E-09	25	-0.0421	0.0478
rs17685	7	75454041	A	G	Exposure1_2	TBV	0.07	0.01	0.29	9.06E-14	49	-0.0056	0.0087
rs2472297	15	72814933	T	C	Exposure1_2	TBV	0.15	0.01	0.24	6.45E-47	225	-0.0066	0.0088
rs4410790	7	17251102	T	C	Exposure1_2	TBV	-0.14	0.01	0.37	1.48E-57	196	0.0068	0.0081
rs7800944	7	72673793	T	C	Exposure1_2	TBV	-0.05	0.01	0.72	7.82E-09	25	-0.0186	0.0086
rs17685	7	75454041	A	G	Exposure1_2	GMV	0.07	0.01	0.29	9.06E-14	49	-0.0038	0.0087
rs2472297	15	72814933	T	C	Exposure1_2	GMV	0.15	0.01	0.24	6.45E-47	225	-0.0198	0.0088
rs4410790	7	17251102	T	C	Exposure1_2	GMV	-0.14	0.01	0.37	1.48E-57	196	0.0121	0.0081
rs7800944	7	72673793	T	C	Exposure1_2	GMV	-0.05	0.01	0.72	7.82E-09	25	-0.0066	0.0086
rs17685	7	75454041	A	G	Exposure1_2	WMV	0.07	0.01	0.29	9.06E-14	49	-0.0054	0.0087
rs2472297	15	72814933	T	C	Exposure1_2	WMV	0.15	0.01	0.24	6.45E-47	225	0.0073	0.0088
rs4410790	7	17251102	T	C	Exposure1_2	WMV	-0.14	0.01	0.37	1.48E-57	196	0.0002	0.0081
rs7800944	7	72673793	T	C	Exposure1_2	WMV	-0.05	0.01	0.72	7.82E-09	25	-0.0220	0.0086
rs17685	7	75454041	A	G	Exposure1_2	left HV	0.07	0.01	0.29	9.06E-14	49	-0.0011	0.0087
rs2472297	15	72814933	T	C	Exposure1_2	left HV	0.15	0.01	0.24	6.45E-47	225	-0.0104	0.0088
rs4410790	7	17251102	T	C	Exposure1_2	left HV	-0.14	0.01	0.37	1.48E-57	196	0.0069	0.0081
rs7800944	7	72673793	T	C	Exposure1_2	left HV	-0.05	0.01	0.72	7.82E-09	25	-0.0138	0.0086
rs17685	7	75454041	A	G	Exposure1_2	right HV	0.07	0.01	0.29	9.06E-14	49	0.0026	0.0087
rs2472297	15	72814933	T	C	Exposure1_2	right HV	0.15	0.01	0.24	6.45E-47	225	-0.0018	0.0088
rs4410790	7	17251102	T	C	Exposure1_2	right HV	-0.14	0.01	0.37	1.48E-57	196	0.0039	0.0081
rs7800944	7	72673793	T	C	Exposure1_2	right HV	-0.05	0.01	0.72	7.82E-09	25	-0.0059	0.0086
rs17685	7	75454041	A	G	Exposure1_2	Any BMB	0.07	0.01	0.29	9.06E-14	49	-0.0097	0.0311
rs2472297	15	72814933	T	C	Exposure1_2	Any BMB	0.15	0.01	0.24	6.45E-47	225	-0.0441	0.0342
rs4410790	7	17251102	T	C	Exposure1_2	Any BMB	-0.14	0.01	0.37	1.48E-57	196	-0.0273	0.0290
rs7800944	7	72673793	T	C	Exposure1_2	Any BMB	-0.05	0.01	0.72	7.82E-09	25	-0.0449	0.0317
rs17685	7	75454041	A	G	Exposure1_2	BMB_lobar	0.07	0.01	0.29	9.06E-14	49	-0.0313	0.0390
rs2472297	15	72814933	T	C	Exposure1_2	BMB_lobar	0.15	0.01	0.24	6.45E-47	225	-0.0419	0.0426
rs4410790	7	17251102	T	C	Exposure1_2	BMB_lobar	-0.14	0.01	0.37	1.48E-57	196	-0.0537	0.0365
rs7800944	7	72673793	T	C	Exposure1_2	BMB_lobar	-0.05	0.01	0.72	7.82E-09	25	-0.0332	0.0394
rs17685	7	75454041	A	G	Exposure1_2	BMB_deep	0.07	0.01	0.29	9.06E-14	49	0.0077	0.0484
rs2472297	15	72814933	T	C	Exposure1_2	BMB_deep	0.15	0.01	0.24	6.45E-47	225	-0.0583	0.0517
rs4410790	7	17251102	T	C	Exposure1_2	BMB_deep	-0.14	0.01	0.37	1.48E-57	196	-0.0076	0.0448
rs7800944	7	72673793	T	C	Exposure1_2	BMB_deep	-0.05	0.01	0.72	7.82E-09	25	-0.0688	0.0478
rs17685	7	75616105	A	G	Exposure2	WMH	0.11	0.02	0.29	4.41E-09	30	0.0074	0.0072
rs2472297	15	75027880	T	C	Exposure2	WMH	0.23	0.02	0.24	1.63E-29	132	0.0039	0.0066
rs6968554	7	17287106	A	G	Exposure2	WMH	-0.23	0.02	0.39	3.13E-38	132	-0.0062	0.0060
rs17685	7	75616105	A	G	Exposure2	FA	0.11	0.02	0.29	4.41E-09	30	0.1177	0.0467
rs2472297	15	75027880	T	C	Exposure2	FA	0.23	0.02	0.24	1.63E-29	132	0.0111	0.0479
rs6968554	7	17287106	A	G	Exposure2	FA	-0.23	0.02	0.39	3.13E-38	132	0.0326	0.0440
rs17685	7	75616105	A	G	Exposure2	MD	0.11	0.02	0.29	4.41E-09	30	-0.0734	0.0478
rs2472297	15	75027880	T	C	Exposure2	MD	0.23	0.02	0.24	1.63E-29	132	0.0441	0.0489
rs6968554	7	17287106	A	G	Exposure2	MD	-0.23	0.02	0.39	3.13E-38	132	0.0069	0.0450
rs17685	7	75616105	A	G	Exposure2	TBV	0.11	0.02	0.29	4.41E-09	30	-0.0056	0.0087
rs2472297	15	75027880	T	C	Exposure2	TBV	0.23	0.02	0.24	1.63E-29	132	-0.0066	0.0088
rs6968554	7	17287106	A	G	Exposure2	TBV	-0.23	0.02	0.39	3.13E-38	132	0.0058	0.0081
rs17685	7	75616105	A	G	Exposure2	GMV	0.11	0.02	0.29	4.41E-09	30	-0.0038	0.0087
rs2472297	15	75027880	T	C	Exposure2	GMV	0.23	0.02	0.24	1.63E-29	132	-0.0198	0.0088
rs6968554	7	17287106	A	G	Exposure2	GMV	-0.23	0.02	0.39	3.13E-38	132	0.0104	0.0081
rs17685	7	75616105	A	G	Exposure2	WMV	0.11	0.02	0.29	4.41E-09	30	-0.0054	0.0087
rs2472297	15	75027880	T	C	Exposure2	WMV	0.23	0.02	0.24	1.63E-29	132	0.0073	0.0088
rs6968554	7	17287106	A	G	Exposure2	WMV	-0.23	0.02	0.39	3.13E-38	132	0.0000	0.0081
rs17685	7	75616105	A	G	Exposure2	left HV	0.11	0.02	0.29	4.41E-09	30	-0.0011	0.0087
rs2472297	15	75027880	T	C	Exposure2	left HV	0.23	0.02	0.24	1.63E-29	132	-0.0104	0.0088
rs6968554	7	17287106	A	G	Exposure2	left HV	-0.23	0.02	0.39	3.13E-38	132	0.0079	0.0081
rs17685	7	75616105	A	G	Exposure2	right HV	0.11	0.02	0.29	4.41E-09	30	0.0026	0.0087
rs2472297	15	75027880	T	C	Exposure2	right HV	0.23	0.02	0.24	1.63E-29	132	-0.0018	0.0088
rs6968554	7	17287106	A	G	Exposure2	right HV	-0.23	0.02	0.39	3.13E-38	132	0.0043	0.0081
rs17685	7	75616105	A	G	Exposure2	Any BMB	0.11	0.02	0.29	4.41E-09	30	-0.0097	0.0311
rs2472297	15	75027880	T	C	Exposure2	Any BMB	0.23	0.02	0.24	1.63E-29	132	-0.0441	0.0342
rs6968554	7	17287106	A	G	Exposure2	Any BMB	-0.23	0.02	0.39	3.13E-38	132	-0.0215	0.0297
rs17685	7	75616105	A	G	Exposure2	BMB_lobar	0.11	0.02	0.29	4.41E-09	30	-0.0313	0.0390
rs2472297	15	75027880	T	C	Exposure2	BMB_lobar	0.23	0.02	0.24	1.63E-29	132	-0.0419	0.0426
rs6968554	7	17287106	A	G	Exposure2	BMB_lobar	-0.23	0.02	0.39	3.13E-38	132	-0.0521	0.0373
rs17685	7	75616105	A	G	Exposure2	BMB_deep	0.11	0.02	0.29	4.41E-09	30	0.0077	0.0484
rs2472297	15	75027880	T	C	Exposure2	BMB_deep	0.23	0.02	0.24	1.63E-29	132	-0.0583	0.0517
rs6968554	7	17287106	A	G	Exposure2	BMB_deep	-0.23	0.02	0.39	3.13E-38	132	0.0048	0.0451

The effects for exposure1 were interpreted as percent change in consumption level per allele.
exposure1: Primary data for cups per day.
exposure1_2: Replication data for cups per day.
exposure2: High coffee consumption vs low/no coffee consumption.

Abbreviations: BMB, brain microbleed; EA, effect allele; FA, fractional anisotropy; HV, hippocampus volume; MD, mean diffusivity; OA, other allele; TBV, total brain volume; SNP, single nucleotide polymorphism; WMH, white matter hyperintensity; WMV; white matter volume.

eTable 2 Bias and Type 1 error rate due to sample overlap

Outcome	SNPs, n	Overlap proportion	Estimate, beta*	Bias	Type 1 error rate
White matter hyperintensity	12	52.6%	0.084	<0.001	0.05
Fractional anisotropy	12	89.8%	0.019	<0.001	0.05
Mean diffusivity	12	89.8%	0.051	<0.001	0.05
Total brain volume	12	9.8%	–0.221	<0.001	0.05
Grey matter volume	12	9.8%	–0.371	<0.001	0.05
White matter volume	12	9.8%	–0.016	<0.001	0.05
Left hippocampus	12	9.8%	–0.135	<0.001	0.05
Right hippocampus	12	9.8%	–0.026	<0.001	0.05
Any cerebral microbleed	12	24.4%	0.113	<0.001	0.05
Cerebral microbleed: mixed or strictly deep	11	24.4%	–0.313	<0.001	0.05
Cerebral microbleed: strictly lobar	11	24.4%	0.263	<0.001	0.05

*The estimate should be in standard deviation units (i.e. per SD increase in the exposure). However, the estimates in our study were in cups per day. Nevertheless, the calculated results showed the biases were negligible.

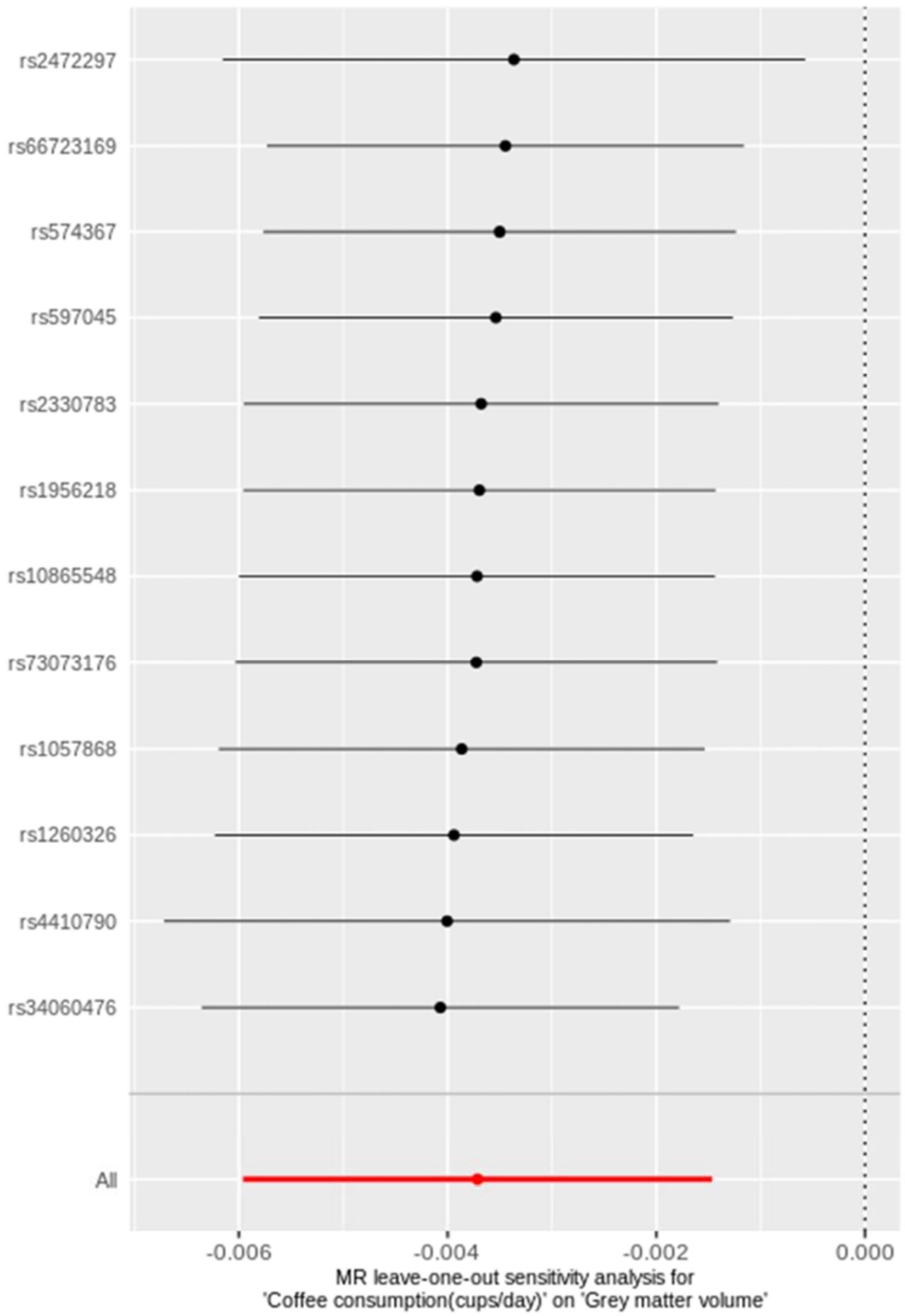

eFigure 1 Leave-one-out plot of coffee consumption (cups/day) on grey matter volume

Outcome	Method	SNPs,n	beta/OR (95% CI)*	p value
White matter hyperintensity	IVW	4	0.053(−0.013, 0.119)	0.117
	Weighted median	4	0.037(−0.026, 0.099)	0.252
	Weighted mode	4	0.036(−0.025, 0.097)	0.327
Fractional anisotropy	IVW	4	0.109(−0.532, 0.750)	0.739
	Weighted median	4	−0.049(−0.517, 0.420)	0.839
	Weighted mode	4	−0.086(−0.555, 0.382)	0.742
Mean diffusivity	IVW	4	0.063(−0.407, 0.533)	0.792
	Weighted median	4	0.128(−0.335, 0.592)	0.588
	Weighted mode	4	0.153(−0.334, 0.640)	0.582
Total brain volume	IVW	4	−0.029(−0.132, 0.074)	0.580
	Weighted median	4	−0.046(−0.127, 0.035)	0.262
	Weighted mode	4	−0.047(−0.128, 0.035)	0.343
Grey matter volume	IVW	4	−0.092(−0.167, −0.018)	0.015
	Weighted median	4	−0.101(−0.186, −0.016)	0.020
	Weighted mode	4	−0.104(−0.197, −0.011)	0.117
White matter volume	IVW	4	0.034(−0.078, 0.146)	0.551
	Weighted median	4	0.020(−0.064, 0.104)	0.639
	Weighted mode	4	0.020(−0.063, 0.103)	0.674
Left hippocampus volume	IVW	4	−0.039(−0.121, 0.044)	0.357
	Weighted median	4	−0.056(−0.140, 0.028)	0.194
	Weighted mode	4	−0.057(−0.139, 0.024)	0.262
Right hippocampus volume	IVW	4	−0.008(−0.083, 0.066)	0.833
	Weighted median	4	−0.018(−0.103, 0.068)	0.687
	Weighted mode	4	−0.018(−0.106, 0.070)	0.709
Any cerebral microbleed	IVW	4	1.008(0.714, 1.424)	0.962
	Weighted median	4	0.939(0.670, 1.316)	0.715
	Weighted mode	4	0.991(0.658, 1.491)	0.966
Cerebral microbleed:	IVW	4	0.954(0.609, 1.493)	0.837
mixed or strictly deep	Weighted median	4	0.928(0.565, 1.524)	0.769
	Weighted mode	4	0.959(0.563, 1.632)	0.887
Cerebral microbleed:	IVW	4	1.058(0.696, 1.609)	0.791
strictly lobar	Weighted median	4	1.040(0.669, 1.617)	0.861
	Weighted mode	4	1.133(0.632, 2.029)	0.704

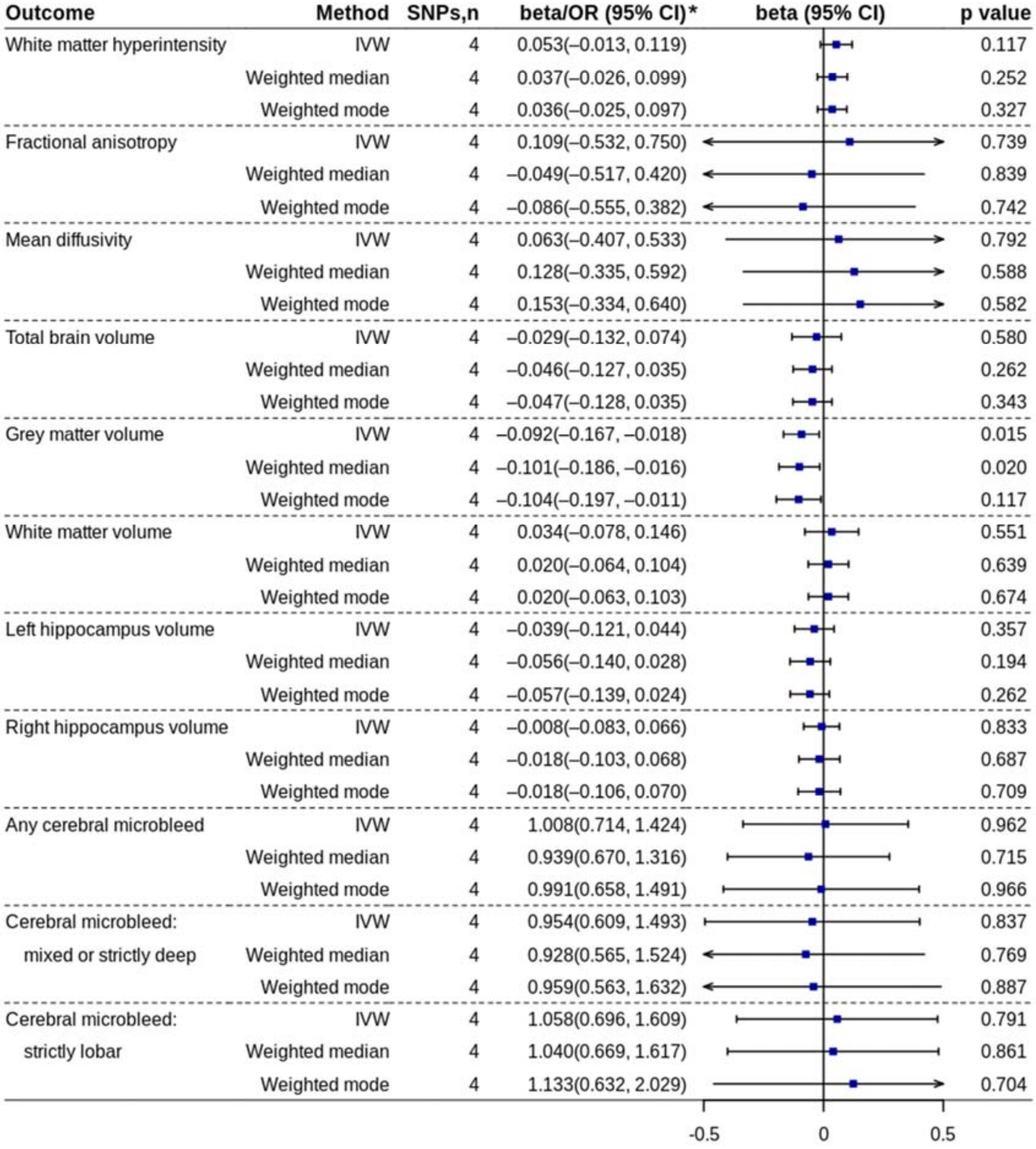

eFigure 2 Replication analyses of coffee consumption (cups/day) on outcomes

*Note ORs with 95% CIs are presented for cerebral microbleed outcomes.

Abbreviations: CI, confidence interval; IVW, inverse variance-weighted; OR, odds ratio; SNP, single nucleotide polymorphism.
